# B7-h3-specific engager T cells for the immunotherapy of pediatric solid tumors

**DOI:** 10.1186/2051-1426-3-S2-P11

**Published:** 2015-11-04

**Authors:** Christopher DeRenzo, David Torres, Victoria Tobin, Phuong Nguyen, Xiao-Tong Song, Stephen Gottschalk

**Affiliations:** 1 Baylor College of Medicine, Houston, TX, USA; 2 Baylor College of Medicine, Center for Cell and Gene Therapy, Department of Pediatrics, Houston, TX, USA

## Background

B7-H3 positive tumors, including osteosarcoma, neuroblastoma, and high grade glioma, cause significant morbidity and mortality despite aggressive multimodality treatments. Current B7-H3-targeted immune-therapies take advantage of the monoclonal antibody 8H9, which is actively being evaluated in Phase I clinical trials. Engager T cells, which secrete bispecific engager molecules consisting of single chain variable fragments specific for CD3 and a tumor antigen, are a new class of antigen-specific T cells, with the unique ability to redirect bystander T cells to tumors, amplifying anti-tumor effects. The goal of this project was to develop B7-H3-specific Engager T cells, and pre-clinically evaluate their effector function *in vitro* and *in vivo*.

## Methods

B7-H3-Engager T cells were generated by transducing T cells with a retroviral vector encoding a B7-H3-specific T cell engager and mOrange separated by an internal ribosomal entry site. B7-H3-Engager T cell effector function was then evaluated *in vitro* and in a metastatic osteosarcoma xenograft model.

## Results

**Figure 1:**
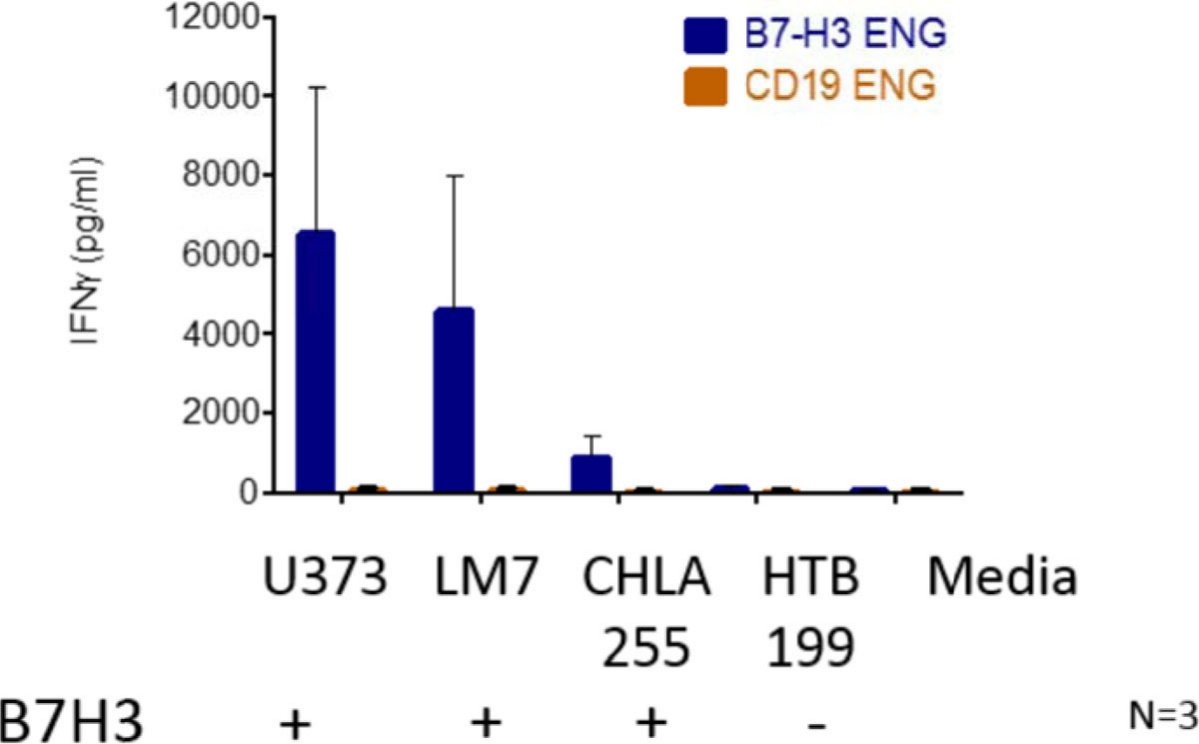


**Figure 2:**
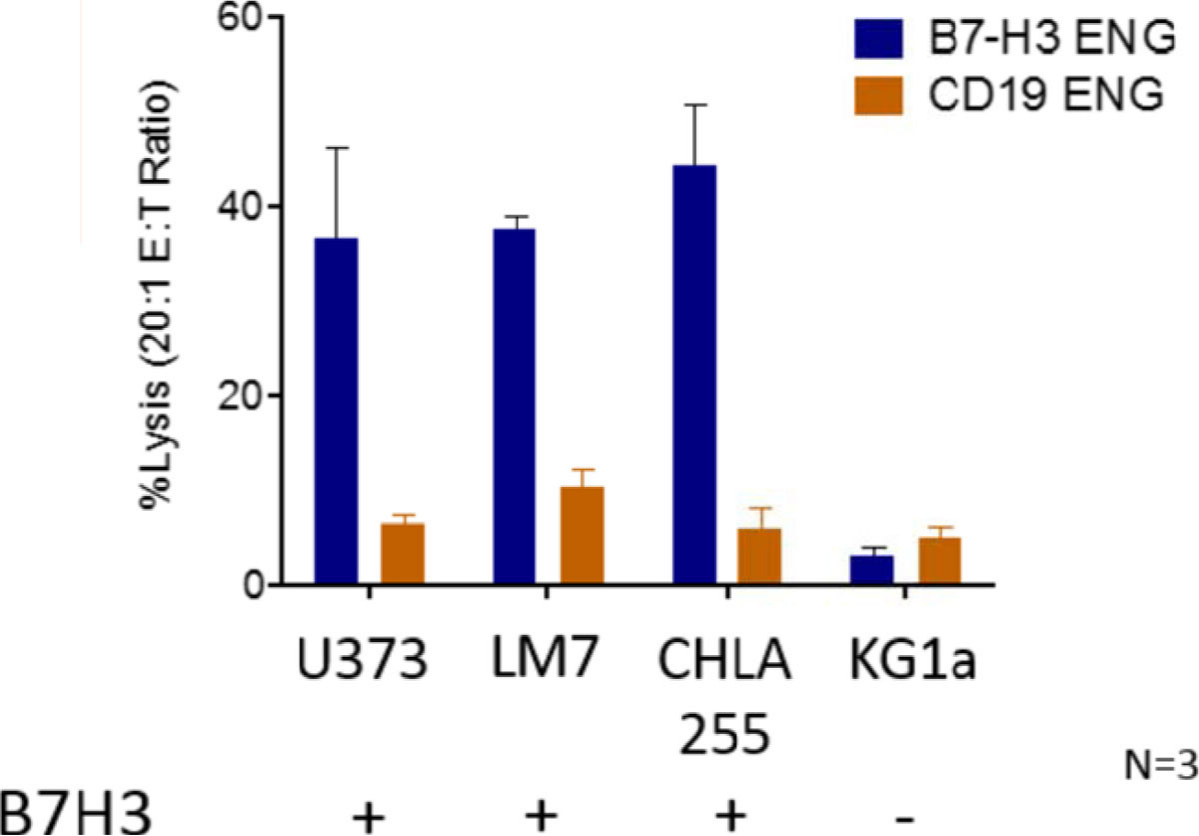


**Figure 3:**
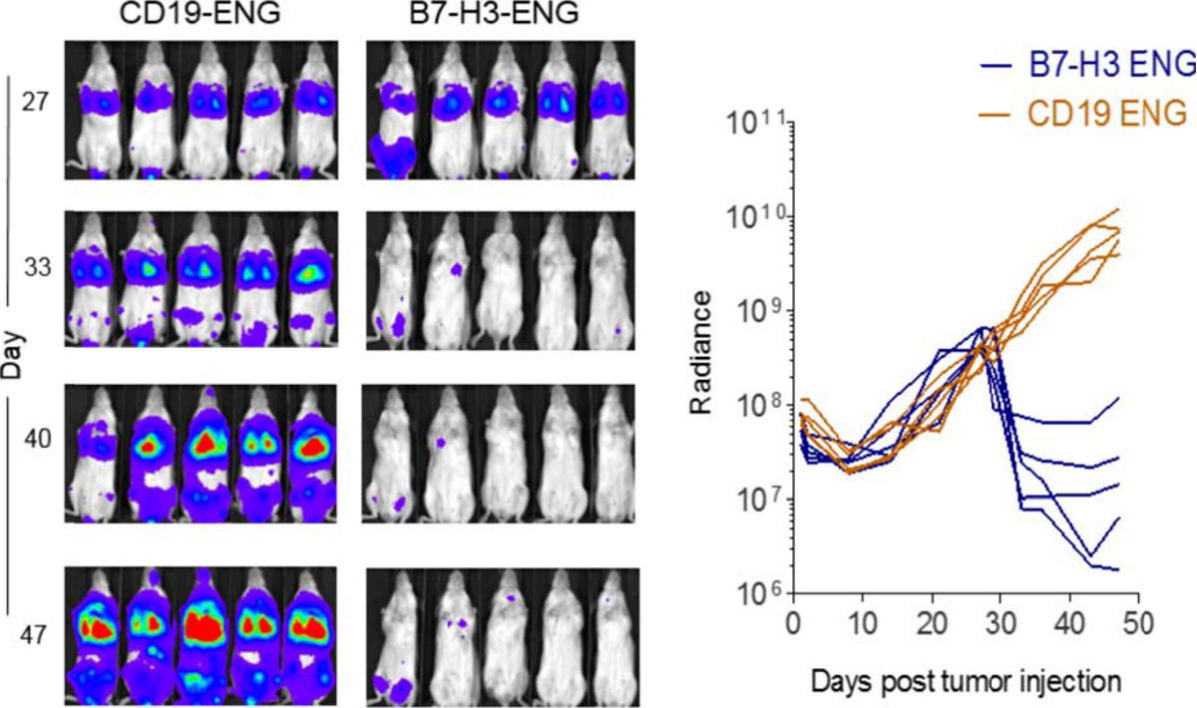


Post transduction 70-84% of T cells were positive for transgene expression. In coculture assay B7-H3-Engager T cells recognized B7-H3-positive osteosarcoma (LM7), neuroblastoma (CHLA255), and glioma (U373) cell lines, as judged by IFN-γ secretion, in contrast to B7-H3-negative tumor cells (HTB-119) (Figure 1). None of the targets were recognized by T cells secreting engager molecules specific for an irrelevant antigen (CD19-Engager T cells). Antigen-dependent recognition was confirmed in standard cytotoxicity assays (Figure 2). To assess anti-tumor activity of B7-H3-Engager T cells *in vivo* we used a metastatic osteosarcoma xenograft model that allows for serial bioluminescence imaging of LM7 cells that are genetically modified to express the fire fly luciferase gene (LM7-ffLuc). NSG mice were intravenously injected with LM7-ffLuc cells, followed by intravenous doses of B7-H3-Engager or control (CD19-Engager) T cells on days 28 and 35 post LM7-ffLuc injection. In contrast to control T cells, B7-H3-Engager T cells had potent anti-osteosarcoma activity (Figure 3) resulting in a survival advantage of treated mice.

## Conclusions

We successfully generated B7-H3-Engager T cells and demonstrate that these cells recognize and kill B7-H3-positive tumor cells in an antigen-dependent manner, and have potent anti-osteosarcoma activity *in vivo*. Thus, B7-H3-Engager T cells may present a promising alternative to current T cell immunotherapy approaches for pediatric solid tumors.

